# Debts and experienced financial scarcity: associations with nonsuicidal self‐injury and suicidality in adolescents at risk for psychopathology

**DOI:** 10.1111/camh.70065

**Published:** 2026-01-22

**Authors:** Susan J. Ravensbergen, Nita G.M. de Neve‐Enthoven, Diandra C. Bouter, Wilco W. van Dijk, Witte J.G. Hoogendijk, Nina H. Grootendorst‐van Mil

**Affiliations:** ^1^ Department of Psychiatry Erasmus MC, University Medical Center Rotterdam Rotterdam The Netherlands; ^2^ Department of Psychiatry iBerry Study Group, Erasmus MC, University Medical Center Rotterdam Rotterdam The Netherlands; ^3^ Knowledge Centre Psychology and Economic Behaviour Leiden The Netherlands; ^4^ Department of Social, Economic and Organisational Psychology Leiden University Leiden The Netherlands; ^5^ Department of Psychiatry Epidemiological and Social Psychiatric Research Institute (ESPRi), Erasmus MC, University Medical Center Rotterdam Rotterdam The Netherlands

**Keywords:** Adolescent, financial stress, suicidal ideation, suicide attempt, self‐injurious behavior, psychopathology

## Abstract

**Background:**

As adolescents transition to increased independence, they may also begin to encounter financial difficulties, including debt, which may contribute to psychological distress. While financial difficulties and experienced financial scarcity have been well‐documented contributors to suicidality in adults, their impact on adolescent populations remains underexplored. The current study aims to elucidate the relationship between late adolescents' own debts or their experienced financial scarcity and the prevalence of self‐harm, including both nonsuicidal self‐injury (NSSI) and suicidality (suicidal ideation/attempt).

**Methods:**

Data from the first follow‐up measurement (T1) of a population‐based high‐risk cohort in the Netherlands were utilized. For the present study, adolescents (*n* = 650, mean age at T1 = 17.9 years) provided self‐reported data on unsecured debts, experienced financial scarcity, NSSI and suicidality. Hierarchical logistic regression analyses were conducted to examine the associations between these variables, adjusting for potential confounding factors, including individual, sociodemographic, and socioenvironmental influences.

**Results:**

The presence of unsecured debts was associated with an increased prevalence of suicidality (adjusted OR = 1.94, *p* = .046), but not with NSSI (*p* = .068). In contrast, greater experienced financial scarcity was associated with a higher prevalence of both NSSI (adjusted OR = 1.09, *p* = .004) and suicidality (adjusted OR = 1.15, *p* < .001).

**Conclusions:**

These results underscore the importance of addressing financial difficulties, particularly debts and experienced financial scarcity, in adolescents at‐risk for NSSI and suicidality. Interventions aimed at mitigating the effects of financial strain – such as financial education or support programs – may be important in reducing the risk of self‐harm in this population. Further research is needed to explore the efficacy of early intervention strategies.

**Key Practitioner Message**

**What is currently known?**

- Adolescence is a vulnerable period for mental health, and while financial difficulties are a known risk factor for suicidality in adults, their impact on adolescent mental health is less understood.

**What has been shown?**

- Experienced financial scarcity is associated with nonsuicidal self‐injury (NSSI).
- Both debts and experienced financial scarcity are linked to higher rates of suicidality (ideations/attempts) in adolescents.

**What is the significance of this for clinical practice?**

- Practitioners should consider financial factors as components in adolescent mental health.
- Early interventions, such as financial education or assistance, may help reduce risks of NSSI and suicidality in this age group.

## Introduction

Financial difficulties have been identified as risk factors for a range of mental health issues, including suicidality (Elbogen et al., 2020; Levinsson et al., 2023; Logan, Hall, & Karch, 2011; Mathieu, Treloar, Hawgood, Ross, & Kõlves, 2022; Meltzer et al., 2011; Naranjo, Glass, & Williams, 2021; Rojas, 2022; Stack & Wasserman, 2007). Suicide is the fourth leading cause of death among 15–29‐year olds globally (WHO, 2023), underscoring the critical need for early identification of associated risk factors. The transition from adolescence into adulthood is a particularly vulnerable period, characterized by numerous developmental challenges (Salmela‐Aro, 2011), including the acquisition of increased autonomy in managing financial responsibilities. The current study investigates associations between financial stressors, specifically debts and experienced financial scarcity, and self‐harm in late adolescence, whereby self‐harm is conceptualized as an overarching term that encompasses both nonsuicidal self‐injury (NSSI) and suicidality, with each representing distinct, yet potentially interrelated, manifestations of psychological distress.

NSSI is characterized by the direct and deliberate destruction of one's own body tissue in the absence of lethal intent (Nock, 2010). Common methods of NSSI include cutting, hitting or banging, and carving, often employed as maladaptive coping strategies for managing negative emotions (Klonsky & Glenn, 2009). Suicidality refers to a spectrum that includes suicidal ideation, plans, and attempts (American Psychological Association, n.d.).

Both NSSI and suicidality commonly arise during adolescence (Nock, 2010) and can be recognized as symptoms of several mental disorders, including mood and anxiety disorders, and borderline personality disorder (American Psychiatric Association, 2013; Bentley et al., 2016; Nock, Joiner, Gordon, Lloyd‐Richardson, & Prinstein, 2006). NSSI and suicidality frequently cooccur, and although NSSI and suicidality have a different motivational background, they can be viewed as related behaviors that may exist on a continuum of self‐harm, with some individuals moving from NSSI to more severe forms of self‐injury, including suicide attempts (Klonsky, Victor, & Saffer, 2014; Ribeiro et al., 2016).

Financial difficulties can encompass a variety of problems as well. Objective financial status may involve debt, while subjective financial stress or experienced financial scarcity refer to the perception of an individual's financial status (Guan, Guariglia, Moore, Xu, & Al‐Janabi, 2022). Debts can be secured or unsecured (loans not backed by collateral) and the latter have been linked to adverse health outcomes (Dote Pardo & Severino‐González, 2025; Fitch, Hamilton, Bassett, & Davey, 2011; Richardson, Elliott, & Roberts, 2013; Ten Have, 2021). Financial difficulties can exacerbate suicidality via factors such as feelings of hopelessness, impaired coping, and diminished self‐worth (Bjørndal, Røysamb, Nes, von Soest, & Ebrahimi, 2025; Frankham, Richardson, & Maguire, 2020). Adolescents' ongoing brain development may also increase risk‐taking and reduce cognitive control compared to adults (Blankenstein, Peper, & Crone, 2022), potentially leading to unwise financial decisions as well as maladaptive coping strategies like self‐harming behaviors, either nonsuicidal or suicidal. Additionally, financial scarcity can have a negative effect on cognitive performance (de Almeida et al., 2024; Mani, Mullainathan, Shafir, & Zhao, 2013) and increase financial avoidance, which involves behavior to avoid dealing with one's financial problems (e.g., avoiding financial information) (Hilbert, Noordewier, & van Dijk, 2022). This can further compromise decision‐making and affect mental health. Ultimately, a vicious circle can occur in which mental health problems can also contribute to extensive buying as a way of self‐soothing (Richardson, Jansen, & Fitch, 2018), or adverse employment outcomes (Gariépy, Danna, Hawke, Henderson, & Iyer, 2022; Rodwell et al., 2018) and lower earnings (Evensen, Lyngstad, Melkevik, Reneflot, & Mykletun, 2017).

In adults, debts and financial difficulties have indeed been related to heightened levels of suicidality and death by suicide (Ali, Md Zabri, Mohd Dasar, & Nordin, 2025; Elbogen et al., 2020; Levinsson et al., 2023; Mathieu et al., 2022; Meltzer et al., 2011; Naranjo et al., 2021; Roelfs & Shor, 2023; Rojas, 2022), with higher odds of death by suicide when debts are present (Ali et al., 2025; Richardson et al., 2013). Within adult men diagnosed with attention‐deficit/hyperactivity disorder, a major increase in debt arrears is observed in the 3 years prior to death by suicide (Beauchaine, Ben‐David, & Bos, 2020). Adolescent‐focused literature is, to the best of our knowledge, nonexistent, except for studies in university populations suggesting a similar relationship (Richardson et al., 2013). A few recent studies do include young adults from the general population, encompassing a broad age span (mainly ranging from 18 years to late 20s). These studies report a general association between financial strain and mental health problems, yet have not specifically addressed suicidality, and mainly included student loan debt only as the primary financial variable (Fan & Ryu, 2023; Kim & Chatterjee, 2021; Sinha, Viswanathan, & Larrison, 2024; Zhang & Kim, 2019). Similarly, few studies address the link between financial difficulties and adolescent NSSI, although research investigating family financial status indicated an increased prevalence of prolonged NSSI among those with a worse financial status (Rajhvajn Bulat, Sušac, & Ajduković, 2024). Studies in adults show a higher incidence of debts in individuals who engage in NSSI (Fitch et al., 2011). The absence of studies on associations between financial problems and self‐harm in adolescence is remarkable, given that both financial problems (including debts) and components of self‐harm (NSSI, suicidal ideation, and suicide attempt) can already emerge before the age of 18 and can be further influenced by important life events in young adulthood (Oksanen, Mikko, & Rantala, 2016; Steinhoff, Bechtiger, Ribeaud, Eisner, & Shanahan, 2020). Furthermore, strong associations with other concerning behaviors, such as criminality and delinquency, have been observed (Hoeve, Jak, Stams, & Meeus, 2014; Hoeve, Stams, et al., 2014).

In summary, most research on associations between financial difficulties and suicidality has predominantly focused on adults, with limited studies examining the impact on NSSI. Since many mental disorders first emerge during adolescence (Solmi et al., 2022), and the transition to adulthood involves increasing financial autonomy, it is critical to study associations between financial difficulties and self‐harm early. Therefore, our study examined the relationship between unsecured debts and experienced financial scarcity, and both NSSI and suicidality in late adolescence while controlling for potentially relevant confounders. We hypothesized that debts and experienced financial scarcity would be associated with an increased prevalence of NSSI and suicidality in adolescents. The confounding variables included age, sex, and ethnic origin, as these demographic factors are known to influence both financial experiences and mental health outcomes (Dote Pardo & Severino‐González, 2025). We additionally adjusted for household monthly income and IQ, due to their established links with susceptibility to psychological stress and financial decision‐making capacity (Callis, Gerrans, Walker, & Gignac, 2023; Janzen & Hellsten, 2021). Importantly, this addressed a key limitation in, for example, prior research in university samples: inadequate control for family socioeconomic status (Richardson et al., 2013). Furthermore, we accounted for social support and parental psychopathology, as both have been consistently associated with adolescent NSSI (Xia, Zhang, & Huang, 2023; Zhou, Liang, Gao, & Liu, 2025) and suicidality (Bratu et al., 2025; Darvishi, Farhadi, & Poorolajal, 2024; Miller, Esposito‐Smythers, & Leichtweis, 2015).

## Methods

### Procedure and participants

This study was part of the iBerry Study, a cohort of 1,022 adolescents from the Greater Rotterdam region, the Netherlands (mean age = 15.0 years, 51.1% female) (Grootendorst‐van Mil et al., 2021). The cohort was oversampled for psychopathology risk using the Strengths and Difficulties Questionnaire‐Youth (van Widenfelt, Goedhart, Treffers, & Goodman, 2003), during routine high school health screenings (mean age at screening = 13.1 years). The top 15% highest scoring adolescents (high‐risk group) and a random sample from the remaining 85% lowest scoring adolescents (low‐risk group) were invited to take part in the iBerry Study, resulting in an oversampling ratio of 2.5:1. Approximately 3 years post‐baseline, 807 adolescents (mean age = 18.1 years, 53.5% female) participated in the first follow‐up (T1, conducted 2019–2022). At both measurements, adolescents and one of their parents attended a visit for a series of structured interviews, questionnaires, cognitive tasks, and physical assessments after providing informed consent. All procedures were clearly explained, ensuring participants fully understood the research procedures, their rights, and their ability to withdraw at any time. Additional details on follow‐up procedures and attrition are provided by Bouter et al. (2024). The Erasmus MC, University Medical Center's Medical Ethics Review Committee approved the study protocol. In the current study, we used data from the T1 measurement, unless otherwise stated. Only data from the questionnaires or cognitive tasks were used.

### Measurements

#### Debts and experienced financial scarcity

In collaboration with the Dutch National Institute for Family Finance Information (Nibud), we developed a questionnaire to evaluate the current personal financial situation of the adolescent (Blanken & der Werf, 2016). Adolescents were asked to indicate whether they had any unsecured financial debts (*yes* or *no*), which included borrowing money from individuals or institutions, payment arrears, bank account overdrafts, and student loans, with the amount of debt in euros. Furthermore, an adapted six‐item version of the Psychological Inventory of Financial Scarcity (PIFS) (van Dijk, van der Werf, & van Dillen, 2022) was administered to assess current experienced financial scarcity. This self‐report questionnaire assesses the adolescents’ perceptions and worries about their financial resources and their sense of control over the financial situation. Items were answered on a 3‐point Likert scale ranging from 0 (*disagree*) to 2 (*agree*). Participant responses on the PIFS were summed into a total score, with higher scores reflecting greater levels of experienced financial scarcity. The reliability and validity of the instrument have been demonstrated (van Dijk et al., 2022). With a Cronbach's alpha of .79, the PIFS had an acceptable internal consistency in our sample.

#### Nonsuicidal self‐injury

The Inventory of Statements about Self‐Injury (ISAS) (Klonsky & Glenn, 2009) is a self‐report measure assessing NSSI behaviors performed intentionally and without suicidal intent. The ISAS measures the frequency of 12 NSSI methods (e.g., cutting, hitting oneself) and includes an open category (Other, namely). Adolescents reported how often they had intentionally engaged in these behaviors in the past 2 years. Responses in the ‘Other’ category were inspected independently by a research psychologist and a psychiatrist and were reassigned if appropriate. Two methods were excluded from further analysis as they did not meet the criteria for NSSI. Specifically, interference with wound healing was excluded based on the Diagnostic and Statistical Manual of Mental Disorders, 5th Edition (American Psychiatric Association, 2013) criteria, which states that disturbance of wound healing or nail biting alone are insufficient to classify as NSSI. Swallowing toxic substances was also excluded, as it was deemed more indicative of suicidal behavior than NSSI (Klonsky, May, & Saffer, 2016).

NSSI was operationalized as engaging in any of the 10 remaining NSSI methods at least once in the past 2 years and dummy‐coded into ‘absent’ (frequency = 0) or ‘present’ (frequency ≥ 1).

#### Suicidality

The 10‐item Questions on Suicide and Self‐Harm—Short Version instrument (VOZZ‐screen) (Kerkhof, Huisman, Vos, & Smits, 2015) was developed to assess the risk of suicidality among adolescents. Each item is rated on a 5‐point Likert scale ranging from 1 to 5, with response options varying across different sections of the questionnaire. This study focuses on suicidal ideations and suicide attempts in the past 2 years, specifically utilizing items 7 to 10. Given the low base rate of suicide attempts and the complete overlap with suicidal ideation in our sample (i.e., all adolescents who reported attempts also reported ideation), we merged suicidal ideation and suicide attempts into a single suicidality variable. This approach increased statistical power by allowing us to analyze suicidality as a broader construct, while acknowledging that ideation and attempts may have partly distinct predictors.

Responses to the four items were summed and total scores subsequently dichotomized, operationalizing suicidality in either ‘absent’ (score = 4) or ‘present’ (scores > 4). The psychometric properties of the VOZZ‐screen are good (Huisman, Smits, & Kerkhof, 2015).

#### Sociodemographic characteristics

Adolescents provided demographic information through a questionnaire on their biological sex at birth, age, and ethnic background. One of the parents reported on the net monthly household income. Information about ethnic background was collected at the baseline and T1 measurement, and classified based on the country of birth of the adolescent and their parents. Answers were dichotomized into Western or non‐Western origin, with a non‐Western origin defined as any country outside Europe, North America, Australia, or New Zealand.

#### Estimated intelligence

Non‐verbal IQ was estimated at the baseline measurement using two subtests (‘Analogies’ and ‘Categories’) from the Snijders‐Oomen Nonverbal Intelligence Test (SON‐R 6–40) (Tellegen & Laros, 2011). Raw subtest scores were combined, doubled, converted into estimated IQ scores using age‐ and sex‐specific norms, and then adjusted for the Flynn effect (Pietschnig & Voracek, 2015; Trahan, Stuebing, Fletcher, & Hiscock, 2014). The SON‐R has shown strong psychometric properties and robust correlations between the ‘Analogies’ and ‘Categories’ subtests and other (verbal) IQ measures such as the WISC‐II/IV (Tellegen & Laros, 2011).

#### Parental psychopathology

The 53‐item Brief Symptom Inventory (BSI) (de Beurs, 2004) is a self‐report questionnaire assessing adult psychological distress and psychiatric symptoms over the past 7 days. Items are answered on a 3‐point scale in the present study and a sum score was created. A higher sum score indicates greater psychological distress or symptom severity. The BSI has demonstrated strong psychometric properties, including high internal consistency and test–retest reliability (Derogatis, 1975). The Cronbach's alpha coefficient in our sample was .92, indicating excellent internal consistency. If the BSI score was missing at the follow‐up assessment, then it was substituted by the baseline BSI score as it reliably indicates the underlying familial vulnerability.

#### Social support

Social support was assessed using the 12‐item Multidimensional Scale of Perceived Social Support (MSPSS) (Zimet, Dahlem, Zimet, & Farley, 1988), which evaluates adolescents’ perceived social support from family, friends, and a significant other. Responses are rated on a 3‐point Likert scale ranging from 0 (*Never or none*) to 2 (*Often or a lot*). A higher sum score reflects greater perceived social support. The MSPSS has been validated and shown to have good reliability in prior studies (Zimet, Powell, Farley, Werkman, & Berkoff, 1990). In the current study, the scale demonstrated good internal consistency, with a Cronbach's alpha coefficient of .86.

### Statistical analyses

#### Analyses

Hierarchical logistic regression analyses were conducted to examine the relationship between debts (dichotomous: no debt vs. any debt) or experienced financial scarcity (continuous) as independent variables and self‐harm outcomes. Given the relatively small sample sizes in cells where debts were the predictor variable, especially in instances where participants reported both debt and NSSI or suicidality, Firth's bias‐reduced logistic regression method was employed to mitigate biases due to small sample size. This method uses a penalized likelihood approach, preventing the model from converging and providing a more stable estimate in small samples (Suhas et al., 2023). Outcomes were subsequently compared with those obtained from the logistic regression analyses to validate consistency of the findings.

The hierarchical regression approach controlled for potential confounders assessed the incremental impact of different covariates. In Model 1, we adjusted for sex and age to account for basic demographic influences. In Model 2, we added ethnic background, IQ score, and parental psychopathology to control for broader sociodemographic and cognitive factors. In Model 3, we included household income to evaluate whether family financial status could account for associations with self‐harm. In Model 4, the final model, we added perceived social support to assess the role of social networks in these relationships. *P*‐values <.05 were considered statistically significant, with all tests being two‐tailed.

Firth's biased‐reduced logistic regressions were performed using R version 4.3.2 with the ‘logistf’ package (version 1.26.0) (Heinze et al., 2023; Puhr, Heinze, Nold, Lusa, & Geroldinger, 2017; Team, 2023). Other analyses were performed using IBM SPSS statistics version 28.0.1.0 (IBM Corp, 2021).

The analytical code used for this study is provided in Appendix S1.

#### Missing data

The study sample comprised adolescents who completed at least one financial and self‐harm measure. Missing items in continuous sum scores (PIFS, BSI, and MSPSS) were addressed using mean imputation, allowing for up to 25% missing data per participant within a variable (within the final complete case sample, missing items were addressed in this manner for 0.6% of the adolescents on the PIFS, 2.3% on the BSI, and 1.2% on the MSPSS). Covariates with missing data included ethnic background (0.1%), household income (5.7%), IQ score (3.7%), perceived social support (10.1%), and parental BSI score (6.5%). The complete case sample (*n* = 650) differed from those with missing data on one of the exposures (debts) and sociodemographic characteristics (see Table S1). The main reason for missing data in covariates appeared to stem from nonparticipating parents, indicating that this may involve different parents or families. Additionally, debts were significantly more prevalent in the group with missing data. Therefore, the missing at random assumption might have been violated, and without sufficient auxiliary variables, applying multiple imputations could introduce result bias. As Hughes et al. describe, complete case analyses would be preferred in this situation and will likely give unbiased estimates (Hughes, Heron, Sterne, & Tilling, 2019). Consequently, analyses were conducted on the complete case sample.

## Results

At T1, a total of 108 adolescents (16.6%) in this study reported NSSI as well as suicidality in the past 2 years. Table 1 presents the characteristics of adolescents for the total sample, as well as stratified by NSSI (*n* = 184, 28.3%) and suicidality (*n* = 158, 24.3%) status. The majority of the sample was female (54.8%), with an even higher representation in the NSSI (66.8%) and suicidality (66.5%) subgroups. Moreover, 85.5% of the participants reported having a Western ethnic background. The household net monthly income primarily fell in the modal range of €2,400–4,399. Debts were reported by 49 adolescents (7.6%, *Mdn* debt = 500.00 euros, *IQR* = 1,373.25), with almost half of them (46.9%) indicating a student loan. Other most frequently reported debt types were borrowing money from others (18.4%), falling behind with payments (8.2%), and mobile phone costs (8.2%). The rank biserial correlation revealed a weak association between debts and experienced financial scarcity (Spearman's *ρ* = .161, *p* < .001).

**Table 1:** Characteristics of the study sample

	Total sample (*n* = 650)	NSSI (*n* = 184)	Suicidality (*n* = 158)
Age (*M*, *SD*)	17.88 (0.72)	17.89 (0.65)	17.90 (0.72)
Sex (*n*, %)			
Male	294 (45.2)	61 (33.2)	53 (33.5)
Female	356 (54.8)	123 (66.8)	105 (66.5)
Ethnic origin (*n*, %)			
Western	556 (85.5)	157 (85.3)	133 (84.2)
Non‐Western	94 (14.5)	27 (14.7)	25 (15.8)
Household net monthly income (*n*, %)			
≤ €1,599	42 (6.5)	13 (7.1)	12 (7.6)
€1,600–2,399	84 (12.9)	28 (15.2)	23 (14.6)
€2,400–4,399	288 (44.3)	72 (39.1)	69 (43.7)
≥ €4,400	236 (36.3)	71 (38.6)	54 (34.2)
IQ score (*M*, *SD*)	99.81 (13.53)	100.23 (13.49)	100.51 (14.63)
Perceived social support (*Mdn*, *IQR*)	20.00 (7.00)	19.00 (7.00)	18.00 (7.00)
Parental psychopathology (*Mdn*, *IQR*)	5.00 (9.00)	6.00 (10.00)	6.50 (11.00)
In debt (*n*, %)	49 (7.6)	20 (10.9)	19 (12.0)
Experienced financial scarcity (*Mdn*, *IQR*)	2.00 (4.00)	3.00 (4.00)	3.00 (5.00)

Missing data: financial debts (*n* = 2) and experienced financial scarcity (*n* = 1). *IQR*, interquartile range; *M*, mean; *Mdn*, median; NSSI, nonsuicidal self‐injury; *SD*, standard deviation.

### Debts and self‐harm

Findings from Firth's logistic regression analyses showed that adolescents in debt initially had 1.87 times higher odds (*p* = .046) of reporting NSSI compared to their nonindebted peers after controlling for age and sex. Yet, this association became nonsignificant after further adjustment for ethnic background, IQ score, and parental psychopathology in Model 2.

The relationship between debts and suicidality followed a different pattern. When controlling for age and sex, adolescents in debt had 2.08 times higher odds (*p* = .022) of experiencing suicidality compared to those without debt (see Table 2). This association persisted after additionally adjusting for all other confounders (adjusted OR = 1.94, *p* = .046).

**Table 2:** Associations of financial factors with nonsuicidal self‐injury and suicidality

	NSSI		Suicidality	
	OR [95% CI]	*p*‐value	OR [95% CI]	*p*‐value
Debts – adjusted for age and sex	**1.87 [1.01; 3.41]**	.**046**	**2.08 [1.11; 3.81]**	.**022**
Debts – additionally adjusted for general confounders^a^	1.85 [0.99; 3.38]	.053	**2.00 [1.06; 3.71]**	.**033**
Debts – additionally adjusted for household income	1.85 [1.00; 3.38]	.052	**1.99 [1.06; 3.68]**	.**034**
Debts – additionally adjusted for social support	1.81 [0.96; 3.36]	.068	**1.94 [1.01; 3.66]**	.**046**
Experienced FS – adjusted for age and sex	**1.11 [1.05; 1.18]**	**<.001**	**1.17 [1.10; 1.24]**	**<.001**
Experienced FS – additionally adjusted for general confounders^a^	**1.11 [1.05; 1.18]**	**<.001**	**1.17 [1.10; 1.24]**	**<.001**
Experienced FS – additionally adjusted for household income	**1.11 [1.05; 1.18]**	**<.001**	**1.17 [1.10; 1.25]**	**<.001**
Experienced FS – additionally adjusted for social support	**1.09 [1.03; 1.16]**	.**004**	**1.15 [1.08; 1.23]**	**<.001**

Significant results are printed in bold. CI, confidence interval; FS, financial scarcity; NSSI, nonsuicidal self‐injury; OR, odds ratio.

^a^ General confounders: ethnic background, IQ score, and parental psychopathology.

Table S2 presents the odds ratios for all variables included in the models.

### Experienced financial scarcity and self‐harm

Logistic regression analyses controlling for age and sex revealed an association between experienced financial scarcity and both NSSI and suicidality (see Table 2). These relationships persisted after adjusting for all other confounders. Specifically, each 1‐point increase in the experienced financial scarcity score was associated with a 9% increase in odds of NSSI (adjusted OR = 1.09, *p* = .004), and a 15% increase in odds of suicidality (adjusted OR = 1.15, *p* < .001).

For the odds ratios of all variables in the models, see Table S2.

## Discussion

The present study is one of the first to study the associations between financial difficulties, specifically debts and experienced financial scarcity, and the prevalence of NSSI and suicidality in late adolescence. Adolescents with unsecured debts had nearly twice the odds of experiencing suicidality compared to their debt‐free peers. Greater experienced financial scarcity was associated with a higher prevalence of both NSSI and suicidality.

The association between debts and an increased incidence of suicidality aligns with findings from adult populations (Ali et al., 2025; Levinsson et al., 2023; Meltzer et al., 2011; Naranjo et al., 2021; Richardson et al., 2013; Rojas, 2022), although the associations were sometimes less pronounced. This could be attributed to the focus on different suicidality‐related factors; adult studies focused predominantly on persons that died by suicide, whereas our study considered a broader spectrum of suicidality by combining suicidal ideations and suicide attempts. Additionally, the relatively low debt levels reported by adolescents suggest that the relationship between debts and suicidality may strengthen with age, as debts have more time to accumulate and negatively impact various aspects of life. On the other hand, as the income of adolescents is generally lower than that of adults, the debt‐income proportion might be equal to or even higher than that of adults. Longitudinal studies are warranted to gain insight into the magnitude of the relationship with suicidality during various life stages. It is noteworthy that almost half of the debts reported by adolescents in our study were student loans, which could be considered an investment, as higher levels of education can lead to higher earnings in the future (Bartholomae et al., 2019). In recent years, the Netherlands has seen a significant rise in student debt, prompting concerns about the risk of students borrowing excessively. This is particularly troubling as outstanding student loans can affect students’ disposable income for as long as 35 years (van der Werf, van Dijk, Schonewille, van der Steeg, & van Dillen, 2022). Our findings suggest that even these more socially accepted forms of debt are related to poorer mental health, potentially contributing to the psychological burden that heightens the risk of suicidality and death by suicide. Furthermore, student loans might serve as a ‘gateway’ debt, fostering patterns of overspending, overborrowing, and escalating debt levels (Achtziger, 2022). This underscores the importance of recognizing all forms of debt as potential stressors, regardless of their perceived severity or social acceptability.

The disappearance of an association between debts and NSSI after adjusting for a broader range of confounders beyond age and sex was unexpected, particularly in light of previous findings demonstrating this relationship in adult populations (Fitch et al., 2011). Given the frequent co‐occurrence of NSSI and suicidality (Klonsky et al., 2014; Ribeiro et al., 2016), one would expect similar associations between both forms of self‐harm and debts. In our sample, the lack of association between debts and NSSI after adjusting for confounders beyond age and sex may be attributable to a power issue, as the sample size may have been insufficient to detect a significant effect. Our findings may suggest that the relationship between debts and NSSI could also be more pronounced with age, which is consistent with our nearly significant odds ratios. Alternatively, it is possible that, in contrast to suicidality, NSSI may not be directly influenced by debts, or that other psychosocial or environmental factors may play a more critical role in the etiology of NSSI.

The association between experienced financial scarcity and both NSSI and suicidality underscores the importance of perceived financial strain as a stressor. The associations are consistent with few previous findings (Mathieu et al., 2022; Rajhvajn Bulat et al., 2024), but contrast with the absence of a similar relationship between objective debts and NSSI. This discrepancy might suggest that NSSI is more influenced by the perception of financial problems than by the actual financial situation. Adolescents who feel financially insecure, regardless of their debt status, might experience lowered levels of self‐esteem, more feelings of hopelessness, and more coping problems, which are well‐documented risk factors for both NSSI and suicidality (Bjørndal et al., 2025; Frankham et al., 2020). The distinction between subjective and objective financial measures highlights the complexity of financial stressors and their mental health consequences.

### Strengths

A key strength of this study is that it is one of the first to focus on an adolescent population, providing valuable insights into the relationship between financial problems and mental health within this age group. Previous research in the Netherlands has highlighted rising payment issues among young adults (18–25 years) (Stichting BKR, 2021); yet our study expands the scope by specifically addressing the mental health impact of financial stressors in adolescents, a group that may be particularly vulnerable but often underrepresented in this type of research. This is particularly relevant given that adolescents may experience unique vulnerabilities, as they navigate developmental changes and gain increasing independence.

We adjusted for a range of individual, sociodemographic, and socioenvironmental covariates to account for potential confounding factors in the analyses. Another strength is our use of both objective and subjective measures of financial problems, coupled with detailed questionnaires assessing NSSI and suicidality. It is also important to acknowledge that our study encompasses a high‐risk sample, which allowed us to increase statistical power. Moreover, the population‐based approach enhanced the generalizability of the findings to a broader population beyond clinical samples (Hauner, Zinbarg, & Revelle, 2014). Additionally, by examining self‐harm across its full spectrum during adolescence, our research offers important insights at a critical stage in development, where early intervention strategies can be especially effective.

### Limitations

Despite efforts to mitigate potential biases, our study has some limitations. Debts reported by the participants mainly concerned student loans, which may limit the generalizability of the findings to other forms of debts. This specific type of debt may not fully represent the broader spectrum of financial liabilities individuals face. Also, we used the complete case sample for analyses, as these adolescents differed from those with incomplete data. Since missingness appeared to be related to the parent not participating, the findings might not be generalizable to adolescents who, for example, have less parental supervision. Another limitation is that although previous studies suggest that financial difficulties increase the risk of self‐harm, we were unable to make any causal inferences. Specifically, the adolescents reported on their current debt status and experienced financial scarcity, while they reported on NSSI and suicidality retrospectively over the past 2 years. Therefore, self‐harm could have had an onset preceding the occurrence of financial difficulties. Furthermore, although we used a large sample size, oversampled on psychopathological risk, and performed statistical analyses suitable for small samples, it is possible that there was too little statistical power to detect the specific association between debts and NSSI. As a result, this association was attenuated after adjustment for potential confounders. Another limitation of this research could be its reliance on self‐reported data. Self‐reporting can introduce biases, such as misreporting or recall errors, potentially affecting the accuracy of the results. However, considering that a significant portion of the debt reported consists of unofficial debts (e.g., informal borrowing from friends), the use of register data would also present challenges and not capture the full extent of financial obligations. Nevertheless, these factors should be considered when interpreting the findings.

### Future research

Future research should focus on exploring the longitudinal patterns of debt accumulation and its impact across different life stages in relation to self‐harm. Investigating the age‐related dynamics of financial burdens could provide valuable insights into how debts evolve over time and its long‐term effects on individuals' financial and mental well‐being. Understanding the specific nature of various debt categories that accumulate over time – whether student loans, credit card debts, or mortgages – along with their relative proportions, would help clarify how different financial obligations contribute to financial stress and psychological well‐being.

In addition, future studies should aim to incorporate objective information on debt status, such as credit reports or other verifiable data sources, to complement self‐reported data. This would enhance the accuracy of the findings and provide a more detailed picture of individuals' financial situations and its impact on mental health. Examining cultural or socioeconomic differences in responses to debts and experienced financial scarcity may help identify vulnerable subgroups that are disproportionately affected by financial burdens.

Further studies could also benefit from exploring the intersection of finance‐related stress with other risk factors for self‐harm, such as substance abuse, self‐esteem, or impulsivity. By considering a broader set of factors that often accompany financial hardship, researchers can develop a more comprehensive understanding of how these elements interact to increase the likelihood of self‐harm.

Finally, while we considered analyzing suicidal ideation and behavior separately, all participants reporting suicide attempts also reported ideation. This overlap supports their conceptual link, though we acknowledge their predictors may differ. Due to low statistical power – particularly the low rate of reported attempts – we could not model them separately in a robust way. Studying ideation and attempts as distinct outcomes remains important, as not all adolescents with suicidal thoughts progress to suicidal behavior, and different risk and protective factors may underlie the transition from ideation to action. Understanding these differential pathways can be critical for prevention and intervention. We therefore recommend that future research with larger or longitudinal samples further disentangle these dimensions.

## Conclusion

These findings emphasize the importance of considering both financial and psychological factors when assessing self‐harm risk among adolescents. Clinicians should consider not only objective financial measures, such as debt amounts or income levels, but also subjective experiences of financial strain. Debts or financial challenges might feel overwhelming to adolescents and could contribute to emotional distress, thereby increasing the risk of self‐harm in its various forms, including NSSI as well as suicidal ideation and suicide attempts. The persistent association between financial difficulties and self‐harm highlights the need for targeted interventions. These should not only address the mental health aspects of self‐harm but also include support for navigating financial difficulties. This may involve financial counseling, debt management strategies, or financial literacy programs. By addressing both economic and psychological challenges, clinicians can better help mitigate the risk of self‐harm in vulnerable populations. Routine screening for financial strain in mental health care could further help identify at‐risk individuals. Although our findings are observational and causality has not been established, a holistic approach that integrates financial and mental health support may be beneficial to reduce self‐harm risk in adolescents facing financial hardship.

## Conflict of interest statement

The authors have declared that they have no competing or potential conflicts of interest.

## Funding information

The iBerry Study is funded by the Erasmus MC, University Medical Center Rotterdam and the following institutes of mental health care (GGz): Parnassia Psychiatric Institute Antes, GGz Breburg, GGz Delfland, GGz Westelijk Noord‐Brabant, and Yulius. All are participating in the Epidemiological and Social Psychiatric Research Institute (ESPRi), a consortium of academic and nonacademic research groups. The work of SJR is also funded by Stichting tot Steun VCVGZ.

## Ethical considerations

This study was conducted in accordance with the guidelines of the World Medical Associations Declaration of Helsinki. Informed consent was obtained from the adolescents themselves and, where applicable, from their parents or caregivers. The study protocol was approved by the Medical Ethics Review Committee of the Erasmus MC, University Medical Center (MEC 2015‐007, approved 02/07/2015, MEC 2018‐1472, approved 29/11/2018).

## Supporting information


**Appendix S1.** Analytical code.

**Table S1.** Comparison of complete case sample versus sample including adolescents with any missing covariate data.

**Table S2.** Hierarchical regression models including all covariates for associations between financial factors and nonsuicidal self‐injury and suicidality.

## Data Availability

The data that support the findings of this study are available on request from the corresponding author. The data are not publicly available due to privacy and ethical restrictions. Other researchers are welcome to collaborate with researchers in the iBerry Study group and to request access to the data. Proposals to collaborate will be assessed by the iBerry Study group with respect to quality, feasibility, and potential overlap with planned or published publications.
