# Supplementary material for: Debts and experienced financial scarcity: associations with nonsuicidal self‐injury and suicidality in adolescents at risk for psychopathology
Source: Child Adolesc Ment Health. 2026 Jan 22;31(3):198–207. doi: 10.1111/camh.70065 (PMC13474302; doi:10.1111/camh.70065)
Supplement: Supplementary file 1 [file CAMH-31-198-s001.pdf]

Table S1. Comparison of complete case sample versus sample including adolescents with any missing covariate data.

	Complete case (*n*=650)	Missing data in covariate(s) (*n*=139)	
Age (*M, SD*)	17.88 (0.72)	18.90 (0.94)	**t=12.08, p<.001**
Sex (*n*, %)			χ^2=2.44, p=.118
Male	294 (45.2)	73 (52.5)	
Female	356 (54.8)	66 (47.5)	
Ethnic background (*n*, %)			**χ^2=23.92, p<.001**
Western	556 (85.5)	94 (68.1)	
Non-Western	94 (14.5)	44 (31.9)	
Household net monthly income (*n*, %)			**χ^2=12.64, p=.005**
≤€1599	42 (6.5)	13 (13.8)	
€1600-2399	84 (12.9)	19 (20.2)	
€2400-4399	288 (44.3)	39 (41.5)	
≥€4400	236 (36.3)	23 (24.5)	
IQ score (*M*, *SD*)	99.81 (13.53)	93.63 (12.66)	**t=-4.48, p<.001**
Perceived social support (*Mdn, IQR*)	20.00 (7.00)	18.00 (8.00)	U=20803.00, p=.278
Parental psychopathology (*Mdn*, *IQR*)	5.00 (9.00)	5.50 (11.75)	U=27328.50, p=.497
In debt (*n*, %)	49 (7.6)	27 (19.6)	**χ^2=18.77, p<.001**
Experienced FS (*Mdn*, *IQR*)	2.00 (4.00)	2.00 (4.00)	U=46813.00, p=.475
NSSI (*n*, %)	184 (28.3)	46 (34.6)	χ^2=2.10, p=.147
Suicidality (*n*, %)	158 (24.3)	37 (26.6)	χ^2=0.33, p=.566

Note. Significant results are printed in bold.
Abbreviations: FS=Financial scarcity; *IQR*=Interquartile range; *M*=Mean; *Mdn*=Median; NSSI=Nonsuicidal self-injury; *SD*=Standard deviation.
