# Supplementary material for: Debts and experienced financial scarcity: associations with nonsuicidal self‐injury and suicidality in adolescents at risk for psychopathology
Source: Child Adolesc Ment Health. 2026 Jan 22;31(3):198–207. doi: 10.1111/camh.70065 (PMC13474302; doi:10.1111/camh.70065)
Supplement: Supplementary file 2 [file CAMH-31-198-s002.pdf]

Table S2. Hierarchical regression models including all covariates for associations between financial factors and nonsuicidal self-injury and suicidality.

		NSSI		Suicidality	
Model		OR [95%CI]	*p*-value	OR [95%CI]	*p*-value
1	Debts	**1.87 [1.01;3.41]**	**.046**	**2.08 [1.11;3.81]**	**.022**
	Age, years	0.94 [0.74;1.20]	.627	1.03 [0.80;1.33]	.792
	Sex, female	**1.98 [1.39;2.84]**	**<.001**	**1.88 [1.29;2.74]**	**<.001**
2	Debts	1.85 [0.99;3.38]	.053	**2.00 [1.06;3.71]**	**.033**
	Age, years	0.95 [0.74;1.21]	.675	1.04 [0.80;1.35]	.742
	Sex, female	**1.95 [1.37;2.79]**	**<.001**	**1.78 [1.22;2.60]**	**.002**
	Ethnic background, non-Western	1.00 [0.60;1.63]	.989	1.09 [0.64;1.80]	.747
	IQ score	1.00 [0.99;1.02]	.698	1.01 [0.99;1.02]	.331
	Parental psychopathology	1.01 [0.99;1.03]	.421	**1.03 [1.01;1.05]**	**.002**
3	Debts	1.85 [1.00;3.38]	.052	**1.99 [1.06;3.68]**	**.034**
	Age, years	0.93 [0.72;1.19]	.585	1.05 [0.81;1.35]	.727
	Sex, female	**1.93 [1.35;2.78]**	**<.001**	**1.77 [1.21;2.59]**	**.003**
	Ethnic background, non-Western	0.98 [0.58;1.64]	.948	1.10 [0.63;1.87]	.726
	IQ score	1.00 [0.99;1.02]	.695	1.01 [0.99;1.02]	.335
	Parental psychopathology	1.01 [0.99;1.03]	.474	**1.03 [1.01;1.05]**	**.003**
	Household net monthly income, €1600-2399	1.06 [0.47;2.44]	.895	1.12 [0.48;2.70]	.801
	Household net monthly income, €2400-4399	0.77 [0.36;1.68]	.500	1.08 [0.50;2.47]	.843
	Household net monthly income, ≥€4400	1.02 [0.47;2.27]	.964	1.06 [0.48;2.48]	.888
4	Debts	1.81 [0.96;3.36]	.068	**1.94 [1.01;3.66]**	**.046**
	Age, years	0.91 [0.70;1.17]	.451	1.02 [0.78;1.33]	.878
	Sex, female	**2.10 [1.46;3.06]**	**<.001**	**1.94 [1.32;2.87]**	**<.001**
	Ethnic background, non-Western	0.86 [0.50;1.46]	.588	0.95 [0.53;1.64]	.857
	IQ score	1.00 [0.99;1.02]	.801	1.01 [0.99;1.02]	.427
	Parental psychopathology	1.00 [0.98;1.02]	.947	**1.02 [1.00;1.05]**	**.023**
	Household net monthly income, €1600-2399	1.07 [0.47;2.52]	.872	1.12 [0.47;2.77]	.806
	Household net monthly income, €2400-4399	0.83 [0.39;1.85]	.639	1.21 [0.54;2.82]	.649
	Household net monthly income, ≥€4400	1.15 [0.53;2.62]	.722	1.22 [0.54;2.93]	.635
	Perceived social support	**0.92 [0.88;0.95]**	**<.001**	**0.91 [0.87;0.94]**	**<.001**
1	Experienced financial scarcity	**1.11 [1.05;1.18]**	**.001**	**1.16 [1.10;1.24]**	**<.001**
	Age, years	0.94 [0.74;1.21]	.637	1.03 [0.80;1.34]	.803
	Sex, female	**1.96 [1.37;2.81]**	**<.001**	**1.85 [1.26;2.71]**	**.002**
2	Experienced financial scarcity	**1.11 [1.05;1.18]**	**<.001**	**1.17 [1.10;1.25]**	**<.001**
	Age, years	0.96 [0.75;1.23]	.751	1.06 [0.82;1.39]	.647
	Sex, female	**1.94 [1.35;2.78]**	**<.001**	**1.77 [1.20;2.60]**	**.004**
	Ethnic background, non-Western	0.98 [0.59;1.62]	.930	1.03 [0.61;1.74]	.918
	IQ score	1.01 [0.99;1.02]	.358	1.01 [1.00;1.03]	.087
	Parental psychopathology	1.01 [0.99;1.03]	.533	**1.03 [1.01;1.05]**	**.004**

3	Experienced financial scarcity	**1.11 [1.05;1.18]**	**<.001**	**1.17 [1.10;1.25]**	**<.001**
	Age, years	0.95 [0.73;1.22]	.670	1.07 [0.82;1.39]	.632
	Sex, female	**1.93 [1.34;2.78]**	**<.001**	**1.77 [1.20;2.60]**	**.004**
	Ethnic background, non-Western	0.96 [0.57;1.63]	.881	1.06 [0.61;1.84]	.841
	IQ score	1.01 [0.99;1.02]	.354	1.01 [1.00;1.03]	.091
	Parental psychopathology	1.01 [0.99;1.03]	.569	**1.03 [1.01;1.05]**	**.005**
	Household net monthly income, €1600-2399	1.08 [0.46;2.50]	.862	1.15 [0.47;2.80]	.765
	Household net monthly income, €2400-4399	0.79 [0.36;1.73]	.561	1.16 [0.51;2.62]	.727
	Household net monthly income, ≥€4400	1.07 [0.48;2.38]	.862	1.16 [0.50;2.69]	.734
4	Experienced financial scarcity	**1.09 [1.03;1.16]**	**.004**	**1.15 [1.08;1.23]**	**<.001**
	Age, years	0.92 [0.71;1.20]	.547	1.04 [0.80;1.37]	.753
	Sex, female	**2.10 [1.44;3.05]**	**<.001**	**1.91 [1.29;2.85]**	**.001**
	Ethnic background, non-Western	0.85 [0.49;1.47]	.565	0.92 [0.52;1.64]	.774
	IQ score	1.01 [0.99;1.02]	.468	1.01 [1.00;1.03]	.140
	Parental psychopathology	1.00 [0.98;1.02]	.999	**1.03 [1.00;1.05]**	**.024**
	Household net monthly income, €1600-2399	1.11 [0.47;2.63]	.811	1.19 [0.47;2.97]	.716
	Household net monthly income, €2400-4399	0.86 [0.39;1.91]	.713	1.30 [0.56;3.02]	.544
	Household net monthly income, ≥€4400	1.21 [0.54;2.73]	.648	1.33 [0.56;3.17]	.521
	Perceived social support	**0.92 [0.89;0.96]**	**<.001**	**0.91 [0.88;0.95]**	**<.001**

Note. Significant results are printed in bold.

Abbreviations: CI=Confidence interval; NSSI=Nonsuicidal self-injury; OR=Odds ratio.
