# Supplementary material for: Debts and experienced financial scarcity: associations with nonsuicidal self‐injury and suicidality in adolescents at risk for psychopathology
Source: Child Adolesc Ment Health. 2026 Jan 22;31(3):198–207. doi: 10.1111/camh.70065 (PMC13474302; doi:10.1111/camh.70065)
Supplement: Supplementary file 3 [file CAMH-31-198-s003.pdf]

Appendix S1. Analytical code

SPSS

** Analyze missings patterns*

```
*subsample.
USE ALL.
COMPUTE filter_sample=((Debts_available = 1 OR PIFS_available = 1) AND (VOZZ_available
=1 OR  ISAS_available =1)).
VARIABLE LABELS  filter_sample '(Debts_available = 1 OR PIFS_available = 1) AND
(VOZZ_available =1 OR  ISAS_available =1) (FILTER)'.
VALUE LABELS  filter_sample 0 'Not Selected' 1 'Selected'.
FORMATS  filter_sample (f1.0).
FILTER BY filter_sample.
EXECUTE.

MULTIPLE IMPUTATION  T1_Debt T1_FI_PIFS_total T1_NSSI T1_VOZZ
Age_T1 Sex Ethnicity_Western SONIQ_C T1_BSI_Total T1_MSPSS_total
T1_income_cat4
/ IMPUTE METHOD = NONE
/ MISSINGSUMMARIES OVERALL VARIABLES (MAXVARS = 100 MINPCTMISSING = 0) PATTERNS.

DO IF
    NOT MISSING(Age_T1)  AND
    NOT MISSING(Sex) AND
    NOT MISSING(Ethnicity_Western) AND
    NOT MISSING(T1_income_cat4) AND
    NOT MISSING(SONIQ_C) AND
    NOT MISSING(T1_BSI_Total) AND
    NOT MISSING(T1_MSPSS_total).
COMPUTE Complete_case = 1.
END IF.
EXE.

DO IF
    (MISSING(Age_T1)  OR
    MISSING(Sex)  OR
    MISSING(Ethnicity_Western) OR
    MISSING(T1_income_cat4)  OR
    MISSING(SONIQ_C) OR
    MISSING(T1_BSI_Total) OR
    MISSING(T1_MSPSS_total)).
COMPUTE Complete_case = 0.
END IF.
```

```
EXE.

FREQUENCIES Complete_case.

T-TEST GROUPS=Complete_case(0 1)
  /MISSING=ANALYSIS
  /VARIABLES=Age_T1
  /ES DISPLAY(TRUE)
  /CRITERIA=CI(.95).
T-TEST GROUPS=Complete_case(0 1)
  /MISSING=ANALYSIS
  /VARIABLES=SONIQ_C
  /ES DISPLAY(TRUE)
  /CRITERIA=CI(.95).
CROSSTABS
  /TABLES=Complete_case BY Sex
  /FORMAT=AVALUE TABLES
  /STATISTICS=CHISQ
  /CELLS=COUNT
  /COUNT ROUND CELL.
CROSSTABS
  /TABLES=Complete_case BY Ethnicity_Western
  /FORMAT=AVALUE TABLES
  /STATISTICS=CHISQ
  /CELLS=COUNT
  /COUNT ROUND CELL.
CROSSTABS
  /TABLES=Complete_case BY T1_income_cat4
  /FORMAT=AVALUE TABLES
  /STATISTICS=CHISQ
  /CELLS=COUNT
  /COUNT ROUND CELL.
CROSSTABS
  /TABLES=Complete_case BY T1_Debt
  /FORMAT=AVALUE TABLES
  /STATISTICS=CHISQ
  /CELLS=COUNT
  /COUNT ROUND CELL.
CROSSTABS
  /TABLES=Complete_case BY T1_VOZZ
  /FORMAT=AVALUE TABLES
  /STATISTICS=CHISQ
  /CELLS=COUNT
  /COUNT ROUND CELL.
CROSSTABS
```

```
  /TABLES=Complete_case BY T1_NSSI
  /FORMAT=AVALUE TABLES
  /STATISTICS=CHISQ
  /CELLS=COUNT
  /COUNT ROUND CELL.

NPTESTS
  /INDEPENDENT TEST (T1_BSI_Total) GROUP (Complete_case)
  /MISSING SCOPE=ANALYSIS USERMISSING=EXCLUDE
  /CRITERIA ALPHA=0.05  CILEVEL=95.
NPTESTS
  /INDEPENDENT TEST (T1_FI_PIFS_total) GROUP (Complete_case)
  /MISSING SCOPE=ANALYSIS USERMISSING=EXCLUDE
  /CRITERIA ALPHA=0.05  CILEVEL=95.
NPTESTS
  /INDEPENDENT TEST (T1_MSPSS_total) GROUP (Complete_case)
  /MISSING SCOPE=ANALYSIS USERMISSING=EXCLUDE
  /CRITERIA ALPHA=0.05  CILEVEL=95.

SORT CASES  BY Complete_case.
SPLIT FILE SEPARATE BY Complete_case.

FREQUENCIES VARIABLES= T1_BSI_Total
  /NTILES=4
  /STATISTICS=RANGE MEDIAN
  /ORDER=ANALYSIS.

FREQUENCIES VARIABLES= T1_FI_PIFS_total
  /NTILES=4
  /STATISTICS=RANGE MEDIAN
  /ORDER=ANALYSIS.

FREQUENCIES VARIABLES= T1_MSPSS_total
  /NTILES=4
  /STATISTICS=RANGE MEDIAN
  /ORDER=ANALYSIS.

FREQUENCIES Sex Ethnicity_Western T1_income_cat4 T1_Debt T1_VOZZ T1_NSSI.
```

** Create complete case sample*

```
USE ALL.
FILTER OFF.
SPLIT FILE OFF.
```

```
COMPUTE filter_sample_compl_case=((filter_sample =1 AND Complete_case =1)).
VARIABLE LABELS  filter_sample_compl_case '(filter_sample =1 AND Complete_case =1)
(FILTER)'.
VALUE LABELS filter_sample_compl_case 0 'Not Selected' 1 'Selected'.
FORMATS  filter_sample_compl_case (f1.0).
FILTER BY filter_sample_compl_case.
EXECUTE.
```

** Calculate Cronbach's alphas*

```
* PIFS.
RELIABILITY
  /VARIABLES=T1_FI_9a T1_FI_9b T1_FI_9c T1_FI_9d T1_FI_9e T1_FI_9f
  /SCALE('ALL VARIABLES') ALL
  /MODEL=ALPHA.
* MSPSS.
RELIABILITY
  /VARIABLES=T1_MSPSS_1 T1_MSPSS_2 T1_MSPSS_3 T1_MSPSS_4 T1_MSPSS_5 T1_MSPSS_6
  T1_MSPSS_7 T1_MSPSS_8 T1_MSPSS_9 T1_MSPSS_10 T1_MSPSS_11 T1_MSPSS_12
  /SCALE('ALL VARIABLES') ALL
  /MODEL=ALPHA.
* BSI.
RELIABILITY
  /VARIABLES= T1_BSI_MO_1_rec T1_BSI_MO_2_rec T1_BSI_MO_3_rec T1_BSI_MO_4_rec
T1_BSI_MO_5_rec T1_BSI_MO_6_rec T1_BSI_MO_7_rec
  T1_BSI_MO_8_rec T1_BSI_MO_9_rec T1_BSI_MO_10_rec T1_BSI_MO_11_rec
T1_BSI_MO_12_rec T1_BSI_MO_13_rec T1_BSI_MO_14_rec T1_BSI_MO_15_rec
  T1_BSI_MO_16_rec T1_BSI_MO_17_rec T1_BSI_MO_18_rec T1_BSI_MO_19_rec
T1_BSI_MO_20_rec T1_BSI_MO_21_rec T1_BSI_MO_22_rec
  T1_BSI_MO_23_rec T1_BSI_MO_24_rec T1_BSI_MO_25_rec T1_BSI_MO_26_rec
T1_BSI_MO_27_rec T1_BSI_MO_28_rec T1_BSI_MO_29_rec T1_BSI_MO_30_rec
  T1_BSI_MO_31_rec T1_BSI_MO_32_rec T1_BSI_MO_33_rec T1_BSI_MO_34_rec
T1_BSI_MO_35_rec T1_BSI_MO_36_rec T1_BSI_MO_37_rec T1_BSI_MO_38_rec
  T1_BSI_MO_39_rec T1_BSI_MO_40_rec T1_BSI_MO_41_rec T1_BSI_MO_42_rec
T1_BSI_MO_43_rec T1_BSI_MO_44_rec T1_BSI_MO_45_rec T1_BSI_MO_46_rec
  T1_BSI_MO_47_rec T1_BSI_MO_48_rec T1_BSI_MO_49_rec T1_BSI_MO_50_rec
T1_BSI_MO_51_rec T1_BSI_MO_52_rec T1_BSI_MO_53_rec
  /SCALE('ALL VARIABLES') ALL
  /MODEL=ALPHA.
```

** Table 1 and other descriptives*

```
* Check distributions of continuous covariates

GRAPH /HISTOGRAM=Age_T1.
GRAPH /HISTOGRAM=SONIQ_C.
GRAPH /HISTOGRAM=T1_BSI_Total.
GRAPH /HISTOGRAM=T1_FI_PIFS_total.
GRAPH /HISTOGRAM=T1_MSPSS_total.

FREQUENCIES Sex Ethnicity_Western T1_income_cat4  T1_Debt  T1_VOZZ T1_NSSI
T1_Debt_type.
* Report mean + SD.
Descriptives Age_T1 SONIQ_C.
* Report median + IQR.
FREQUENCIES VARIABLES= T1_BSI_Total  T1_FI_PIFS_total T1_MSPSS_total T1_Debt_amount
  /FORMAT=NOTABLE
  /NTILES=4
  /STATISTICS= MEDIAN.

SORT CASES  BY T1_VOZZ.
SPLIT FILE LAYERED BY T1_VOZZ.

FREQUENCIES Sex Ethnicity_Western T1_income_cat4  T1_Debt  T1_NSSI T1_Debt_type.
* Report mean + SD.
Descriptives Age_T1 SONIQ_C.
* Report median + IQR.
FREQUENCIES VARIABLES= T1_BSI_Total  T1_FI_PIFS_total T1_MSPSS_total T1_Debt_amount
  /FORMAT=NOTABLE
  /NTILES=4
  /STATISTICS= MEDIAN.

SPLIT FILE OFF.

SORT CASES  BY T1_NSSI.
SPLIT FILE LAYERED BY T1_NSSI.

FREQUENCIES Sex Ethnicity_Western T1_income_cat4  T1_Debt T1_VOZZ T1_Debt_type.
* Report mean + SD.
Descriptives Age_T1 SONIQ_C.
* Report median + IQR.
FREQUENCIES VARIABLES= T1_BSI_Total  T1_FI_PIFS_total T1_MSPSS_total T1_Debt_amount
  /FORMAT=NOTABLE
  /NTILES=4
  /STATISTICS= MEDIAN.

SPLIT FILE OFF.
```

```
* Correlation debt and PIFS score

CORRELATIONS
  /VARIABLES= T1_Debt T1_FI_PIFS_total
  /PRINT=TWOTAIL NOSIG FULL
  /MISSING=PAIRWISE.
NONPAR CORR
  /VARIABLES=T1_Debt T1_FI_PIFS_total
  /PRINT=BOTH TWOTAIL NOSIG FULL
  /MISSING=PAIRWISE.
```

*** Logistic regression analyses**

```
* Financial scarcity – suicidality
LOGISTIC REGRESSION VARIABLES T1_VOZZ
  /METHOD=ENTER T1_FI_PIFS_total Age_T1 Sex
  /METHOD=ENTER Ethnicity_Western SONIQ_C T1_BSI_Total
  /METHOD=ENTER T1_income_cat4
  /METHOD=ENTER T1_MSPSS_total
  /CONTRAST (Sex)=Indicator(1)
  /CONTRAST (Ethnicity_Western)=Indicator(1)
  /CONTRAST (T1_income_cat4)=Indicator(1)
  /CLASSPLOT
  /PRINT=GOODFIT CORR SUMMARY CI(95)
  /CRITERIA=PIN(0.05) POUT(0.10) ITERATE(20) CUT(0.5).

* Financial scarcity – NSSI
LOGISTIC REGRESSION VARIABLES T1_NSSI
  /METHOD=ENTER T1_FI_PIFS_total Age_T1 Sex
  /METHOD=ENTER Ethnicity_Western SONIQ_C T1_BSI_Total
  /METHOD=ENTER T1_income_cat4
  /METHOD=ENTER T1_MSPSS_total
  /CONTRAST (Sex)=Indicator(1)
  /CONTRAST (Ethnicity_Western)=Indicator(1)
  /CONTRAST (T1_income_cat4)=Indicator(1)
  /CLASSPLOT
  /PRINT=GOODFIT CORR SUMMARY CI(95)
  /CRITERIA=PIN(0.05) POUT(0.10) ITERATE(20) CUT(0.5).

* Debt – suicidality > compare with Firth
LOGISTIC REGRESSION VARIABLES T1_VOZZ
  /METHOD=ENTER T1_Debt Age_T1 Sex
  /METHOD=ENTER Ethnicity_Western SONIQ_C T1_BSI_Total
```

```
  /METHOD=ENTER T1_income_cat4
  /METHOD=ENTER T1_MSPSS_total
  /CONTRAST (T1_Debt)=Indicator(1)
  /CONTRAST (Sex)=Indicator(1)
  /CONTRAST (Ethnicity_Western)=Indicator(1)
  /CONTRAST (T1_income_cat4)=Indicator(1)
  /CLASSPLOT
  /PRINT=GOODFIT CORR SUMMARY CI(95)
  /CRITERIA=PIN(0.05) POUT(0.10) ITERATE(20) CUT(0.5).

* Debt - NSSI > compare with Firth
LOGISTIC REGRESSION VARIABLES T1_NSSI
  /METHOD=ENTER T1_Debt Age_T1 Sex
  /METHOD=ENTER Ethnicity_Western SONIQ_C T1_BSI_Total
  /METHOD=ENTER T1_income_cat4
  /METHOD=ENTER T1_MSPSS_total
  /CONTRAST (T1_Debt)=Indicator(1)
  /CONTRAST (Sex)=Indicator(1)
  /CONTRAST (Ethnicity_Western)=Indicator(1)
  /CONTRAST (T1_income_cat4)=Indicator(1)
  /CLASSPLOT
  /PRINT=GOODFIT CORR SUMMARY CI(95)
  /CRITERIA=PIN(0.05) POUT(0.10) ITERATE(20) CUT(0.5).
```

R statistics

* **Firth's regression debts – suicidality**

```
firth_SUIC1 <-logistf(T1_VOZZ~ T1_Debt + Age_T1 + Sex
                                , data = Dataset_R)

summary(firth_SUIC1)

coefficients <- firth_SUIC1$coefficients
std_errors <- sqrt(diag(firth_SUIC1$var))
p_values <- firth_SUIC1$prob
OR <- exp(firth_SUIC1$coefficients)
CI_lower <- exp(firth_SUIC1$ci.lower)
CI_upper <- exp(firth_SUIC1$ci.upper)

summary_table_complete_SUIC1 <- data.frame(
   Coefficient = coefficients,
   `Std. Error` = std_errors,
```

```
  `p-value` = p_values,
  OR = OR,
  `CI Lower` = CI_lower,
  `CI Upper` = CI_upper
)

print(summary_table_complete_SUIC1)


firth_SUIC2<-logistf(T1_VOZZ~ T1_Debt + Age_T1 + Sex +
                     Ethnicity_Western + SONIQ_C + T1_BSI_Total
                     , data = Dataset_R)

coefficients <- firth_SUIC2$coefficients
std_errors <- sqrt(diag(firth_SUIC2$var))
p_values <- firth_SUIC2$prob
OR <- exp(firth_SUIC2$coefficients)
CI_lower <- exp(firth_SUIC2$ci.lower)
CI_upper <- exp(firth_SUIC2$ci.upper)

summary_table_complete_SUIC2 <- data.frame(
  Coefficient = coefficients,
  `Std. Error` = std_errors,
  `p-value` = p_values,
  OR = OR,
  `CI Lower` = CI_lower,
  `CI Upper` = CI_upper
)

print(summary_table_complete_SUIC2)


firth_SUIC3 <-logistf(T1_VOZZ~ T1_Debt + Age_T1 + Sex +
                     Ethnicity_Western + SONIQ_C + T1_BSI_Total +
                     T1_income_cat4
                     , data = Dataset_R)

coefficients <- firth_SUIC3$coefficients
std_errors <- sqrt(diag(firth_SUIC3$var))
p_values <- firth_SUIC3$prob
OR <- exp(firth_SUIC3$coefficients)
CI_lower <- exp(firth_SUIC3$ci.lower)
CI_upper <- exp(firth_SUIC3$ci.upper)

summary_table_complete_SUIC3 <- data.frame(
```

```
    Coefficient = coefficients,
    `Std. Error` = std_errors,
    `p-value` = p_values,
    OR = OR,
    `CI Lower` = CI_lower,
    `CI Upper` = CI_upper
 )

print(summary_table_complete_SUIC3)


firth_SUIC4 <-logistf(T1_VOZZ~ T1_Debt + Age_T1 + Sex +
                            Ethnicity_Western + SONIQ_C + T1_BSI_Total +
                            T1_income_cat4 +
                            T1_MSPSS_total
                            , data = Dataset_R)

coefficients <- firth_SUIC4$coefficients
std_errors <- sqrt(diag(firth_SUIC4$var))
p_values <- firth_SUIC4$prob
OR <- exp(firth_SUIC4$coefficients)
CI_lower <- exp(firth_SUIC4$ci.lower)
CI_upper <- exp(firth_SUIC4$ci.upper)

summary_table_complete_SUIC4 <- data.frame(
    Coefficient = coefficients,
    `Std. Error` = std_errors,
    `p-value` = p_values,
    OR = OR,
    `CI Lower` = CI_lower,
    `CI Upper` = CI_upper
 )

print(summary_table_complete_SUIC4)

summary(firth_SUIC4)
```

* **Firth's regression debts - NSSI**

```
firth_NSSI1 <-logistf(T1_NSSI~ T1_Debt + Age_T1 + Sex
                            , data = Dataset_R)

summary(firth_NSSI1)
```

```
coefficients <- firth_NSSI1$coefficients
std_errors <- sqrt(diag(firth_NSSI1$var))
p_values <- firth_NSSI1$prob
OR <- exp(firth_NSSI1$coefficients)
CI_lower <- exp(firth_NSSI1$ci.lower)
CI_upper <- exp(firth_NSSI1$ci.upper)

summary_table_complete_NSSI1 <- data.frame(
  Coefficient = coefficients,
  `Std. Error` = std_errors,
  `p-value` = p_values,
  OR = OR,
  `CI Lower` = CI_lower,
  `CI Upper` = CI_upper
)

print(summary_table_complete_NSSI1)


firth_NSSI2<-logistf(T1_NSSI~ T1_Debt + Age_T1 + Sex +
                     Ethnicity_Western + SONIQ_C + T1_BSI_Total
                     , data = Dataset_R)

coefficients <- firth_NSSI2$coefficients
std_errors <- sqrt(diag(firth_NSSI2$var))
p_values <- firth_NSSI2$prob
OR <- exp(firth_NSSI2$coefficients)
CI_lower <- exp(firth_NSSI2$ci.lower)
CI_upper <- exp(firth_NSSI2$ci.upper)

summary_table_complete_NSSI2 <- data.frame(
  Coefficient = coefficients,
  `Std. Error` = std_errors,
  `p-value` = p_values,
  OR = OR,
  `CI Lower` = CI_lower,
  `CI Upper` = CI_upper
)

print(summary_table_complete_NSSI2)


firth_NSSI3 <-logistf(T1_NSSI~ T1_Debt + Age_T1 + Sex +
                     Ethnicity_Western + SONIQ_C + T1_BSI_Total +
                     T1_income_cat4
```

```
                                    , data = Dataset_R)

coefficients <- firth_NSSI3$coefficients
std_errors <- sqrt(diag(firth_NSSI3$var))
p_values <- firth_NSSI3$prob
OR <- exp(firth_NSSI3$coefficients)
CI_lower <- exp(firth_NSSI3$ci.lower)
CI_upper <- exp(firth_NSSI3$ci.upper)

summary_table_complete_NSSI3 <- data.frame(
   Coefficient = coefficients,
   `Std. Error` = std_errors,
   `p-value` = p_values,
   OR = OR,
   `CI Lower` = CI_lower,
   `CI Upper` = CI_upper
 )

print(summary_table_complete_NSSI3)


firth_NSSI4 <-logistf(T1_NSSI~ T1_Debt + Age_T1 + Sex +
                                    Ethnicity_Western + SONIQ_C + T1_BSI_Total +
                                    T1_income_cat4 +
                                    T1_MSPSS_total
                                    , data = Dataset_R)

coefficients <- firth_NSSI4$coefficients
std_errors <- sqrt(diag(firth_NSSI4$var))
p_values <- firth_NSSI4$prob
OR <- exp(firth_NSSI4$coefficients)
CI_lower <- exp(firth_NSSI4$ci.lower)
CI_upper <- exp(firth_NSSI4$ci.upper)

summary_table_complete_NSSI4 <- data.frame(
   Coefficient = coefficients,
   `Std. Error` = std_errors,
   `p-value` = p_values,
   OR = OR,
   `CI Lower` = CI_lower,
   `CI Upper` = CI_upper
 )

print(summary_table_complete_NSSI4)
```

```
summary(firth_NSSI4)
```

* **Assumptions logistic regression**

```
fit1 <- with(data = Dataset_R, exp = glm(T1_VOZZ  ~ T1_Debt + Age_T1 + Sex + Ethnicity_
Western + SONIQ_C + T1_BSI_Total + T1_income_cat4 + T1_MSPSS_total + T1_YSR_sip, fami
ly = binomial))

fit2 <- with(data = Dataset_R, exp = glm(T1_NSSI  ~ T1_Debt + Age_T1 + Sex + Ethnicity_W
estern + SONIQ_C + T1_BSI_Total +
T1_income_cat4 + T1_MSPSS_total + T1_YSR_sip, family = binomial))

fit3 <- with(data = Dataset_R, exp = glm(T1_VOZZ  ~ T1_FI_PIFS_total + Age_T1 + Sex + Eth
nicity_Western + SONIQ_C + T1_BSI_Total + T1_income_cat4 + T1_MSPSS_total + T1_YSR_si
p, family = binomial))

fit4 <- with(data = Dataset_R, exp = glm(T1_NSSI  ~ T1_FI_PIFS_total + Age_T1 + Sex + Ethn
icity_Western + SONIQ_C + T1_BSI_Total + T1_income_cat4 + T1_MSPSS_total + T1_YSR_sip,
family = binomial))

vif_values1 <- vif(fit1)
vif_values2 <- vif(fit2)
vif_values3 <- vif(fit3)
vif_values4 <- vif(fit4)

> print(vif_values1)
> print(vif_values2)
> print(vif_values3)
> print(vif_values4)


pearson_chi_square1 <- sum(residuals(fit1, type = "pearson")^2)
df1 <- fit1$df.residual
overdispersion_ratio1 <- pearson_chi_square1 / df1
overdispersion_ratio1

pearson_chi_square2 <- sum(residuals(fit2, type = "pearson")^2)
df2 <- fit2$df.residual
overdispersion_ratio2 <- pearson_chi_square2 / df2
overdispersion_ratio2

pearson_chi_square3 <- sum(residuals(fit3, type = "pearson")^2)
df3 <- fit3$df.residual
overdispersion_ratio3 <- pearson_chi_square3 / df3
```

```
overdispersion_ratio3

pearson_chi_square4 <- sum(residuals(fit4, type = "pearson")^2)
df4 <- fit4$df.residual
overdispersion_ratio4 <- pearson_chi_square4 / df4
overdispersion_ratio4



Dataset_R$log_Age_T1 <- log(Dataset_R$Age_T1)
Dataset_R$interaction_Age_T1 <- Dataset_R$Age_T1 * Dataset_R$log_Age_T1

Dataset_R$log_SONIQ_C <- log(Dataset_R$SONIQ_C)
Dataset_R$interaction_SONIQ_C <- Dataset_R$SONIQ_C * Dataset_R$log_SONIQ_C

Dataset_R$log_T1_BSI_Total <- log(Dataset_R$ T1_BSI_Total)
Dataset_R$interaction_T1_BSI_Total <- Dataset_R$T1_BSI_Total *
Dataset_R$log_T1_BSI_Total

Dataset_R$log_T1_MSPSS_total  <- log(Dataset_R$T1_MSPSS_total)
Dataset_R$interaction_T1_MSPSS_total <- Dataset_R$T1_MSPSS_total  *
Dataset_R$log_T1_MSPSS_total

Dataset_R$log_T1_YSR_sip <- log(Dataset_R$T1_YSR_sip) Dataset_R$interaction_T1_YSR_sip
<- Dataset_R$T1_YSR_sip * Dataset_R$log_T1_YSR_sip

fit1_lin <- with(data = Dataset_R, exp = glm(T1_VOZZ  ~ T1_Debt + Age_T1 + Sex + Ethnici
ty_Western + SONIQ_C + T1_BSI_Total + T1_income_cat4 + T1_MSPSS_total + T1_YSR_sip +
log_Age_T1 + interaction_Age_T1 + log_SONIQ_C + interaction_SONIQ_C + log_T1_BSI_Total
+ interaction_T1_BSI_Total + log_T1_MSPSS_total + interaction_T1_MSPSS_total + log_T1_YS
R_sip + interaction_T1_YSR_sip, family = binomial))

fit2_lin <- with(data = Dataset_R, exp = glm(T1_NSSI  ~ T1_Debt + Age_T1 + Sex + Ethnicit
y_Western + SONIQ_C + T1_BSI_Total +
T1_income_cat4 + T1_MSPSS_total + T1_YSR_sip + log_Age_T1 + interaction_Age_T1 + log_
SONIQ_C + interaction_SONIQ_C + log_T1_BSI_Total + interaction_T1_BSI_Total + log_T1_MS
PSS_total + interaction_T1_MSPSS_total + log_T1_YSR_sip + interaction_T1_YSR_sip, family =
binomial))

fit3_lin <- with(data = Dataset_R, exp = glm(T1_VOZZ  ~ T1_FI_PIFS_total + Age_T1 + Sex +
Ethnicity_Western + SONIQ_C + T1_BSI_Total + T1_income_cat4 + T1_MSPSS_total + T1_YSR
_sip + log_Age_T1 + interaction_Age_T1 + log_SONIQ_C + interaction_SONIQ_C + log_T1_BS
I_Total + interaction_T1_BSI_Total + log_T1_MSPSS_total + interaction_T1_MSPSS_total + log
_T1_YSR_sip + interaction_T1_YSR_sip, family = binomial))
```

```
fit4_lin <- with(data = Dataset_R, exp = glm(T1_NSSI  ~ T1_FI_PIFS_total + Age_T1 + Sex +
Ethnicity_Western + SONIQ_C + T1_BSI_Total + T1_income_cat4 + T1_MSPSS_total + T1_YSR
_sip + log_Age_T1 + interaction_Age_T1 + log_SONIQ_C + interaction_SONIQ_C + log_T1_BS
I_Total + interaction_T1_BSI_Total + log_T1_MSPSS_total + interaction_T1_MSPSS_total + log
_T1_YSR_sip + interaction_T1_YSR_sip, family = binomial))

summary(fit1_lin)
summary(fit2_lin)
summary(fit3_lin)
summary(fit4_lin)
```
